# Direct compression of 170-fs 50-cycle pulses down to 1.5 cycles with 70% transmission

**DOI:** 10.1038/s41598-018-30198-y

**Published:** 2018-08-07

**Authors:** Young-Gyun Jeong, Riccardo Piccoli, Denis Ferachou, Vincent Cardin, Michael Chini, Steffen Hädrich, Jens Limpert, Roberto Morandotti, François Légaré, Bruno E. Schmidt, Luca Razzari

**Affiliations:** 1 Centre Énergie Matériaux Télécommunications, Institut National de la Recherche Scientifique (INRS-EMT), 1650 Boulevard Lionel-Boulet, Varennes, Québec J3X 1S2 Canada; 2 few-cycle Inc., 2890 Rue de Beaurivage, Montréal, Québec H1L 5W5 Canada; 3 Department of Physics and CREOL, University of Central Florida, Orlando, Florida 32816 USA; 4 Active Fiber Systems GmbH, Ernst-Ruska-Ring 11, 07745 Jena, Germany; 5 Institute of Applied Physics, Abbe Center of Photonics, Friedrich-Schiller-University Jena, Albert-Einstein-Str. 15, 07745 Jena, Germany; 6 Fraunhofer Institute for Applied Optics and Precision Engineering, Albert-Einstein-Str. 7, 07745 Jena, Germany; 7 ITMO University, 199034 St. Petersburg, Russia; 8 Institute of Fundamental and Frontier Sciences, University of Electronic Science and Technology of China, Chengdu, 610054 Sichuan China

## Abstract

We present a straightforward route for extreme pulse compression, which relies on moderately driving self-phase modulation (SPM) over an extended propagation distance. This avoids that other detrimental nonlinear mechanisms take over and deteriorate the SPM process. The long propagation is obtained by means of a hollow-core fiber (HCF), up to 6 m in length. This concept is potentially scalable to TW pulse peak powers at kW average power level. As a proof of concept, we demonstrate 33-fold pulse compression of a 1 mJ, 6 kHz, 170 fs Yb laser down to 5.1 fs (1.5 cycles at 1030 nm), by employing a single HCF and subsequent chirped mirrors with an overall transmission of 70%.

## Introduction

Over the last decade, high-energy optical pulses close to single-cycle duration have opened new ground in the investigation of ultrafast and strong-field-driven laser phenomena, such as generation of high-energy isolated attosecond pulses^1^, few-femtosecond electron dynamics in molecules^2^, and waveform control of broadband terahertz radiation^3^, to name but a few. However, the direct generation of such optical pulses is still very challenging, since it requires ultra-broadband spectra exceeding the limits of common laser gain media. Therefore, in order to support operation in the few-cycle regime, the spectra of laser pulses have to be further extended by means of nonlinear effects and recompressed afterwards. In this context, a tremendous energy boost, up to the sub-mJ range, was reached by circumventing bulk nonlinearities through the use of gas-filled HCFs, a technique pioneered by Nisoli *et al*. in 1996^4^. A further step in pulse compression was achieved through the invention of chirped mirrors^5^ and their use for ultra-broadband dispersion control^6^. This technology development peaked with the generation and control of sub-cycle light fields based on HCF broadening^7^.

Despite the large impact of these HCF-based setups for ultrafast science, ionization and self-focusing in the gas still limit the employable pulse energy at the mJ level for a meter-long rigid capillary. Such restraint can be lessened by employing circularly-polarized light or by pre-chirping the input pulses^8^. However, a significant improvement could be only achieved by using considerably longer HCFs^9,10^. Nowadays, pulse energies in the range 5–10 mJ can be obtained at the output of 3-m-long fibers^11,12^. Another striking capability of the HCF concept is its applicability to compress pulses at unprecedentedly high average power levels of hundreds of Watts^13,14^. This high average power compression became possible with the advent of Yb lasers. Such sources outperform the well-established Ti:Sapphire technology on many aspects, with one major exception: the gain bandwidth. While Yb-glass- or Yb-CaF_2_-based systems can reach pulse durations as short as 200 fs^15,16^, the typical duration of multi-mJ Yb-YAG systems lies in the few-picosecond range^17–19^. To obtain pulse durations comparable to Ti:Sapphire lasers, let alone few-cycle pulses, post compression is essential. In recent years, various bulk compression schemes have been developed and applied in the lower energy range below 100 µJ and for moderate compression factors of 5 to 6^20,21^. Very recently, 18-fold compression of an Yb laser has been demonstrated in a single, 1-m-long HCF that however required advanced phase compensation with a pulse shaper for few tens of µJ output energy^22^. Multi-stage setups have also been used to achieve high compression factors at low energies: (i) three-stage setup based on BBO (6-fold compression − 3 µJ, 30 fs, 75% throughput)^23^, (ii) three-stage setup with fused silica (FS) as the nonlinear medium (20-fold compression −2.5 µJ, 10 fs, 70% throughput)^24^, (iii) two-stage setup based on gas filled Kagome fibers (27-fold compression −0.37 µJ, 9 fs, 45% throughput)^25^, (iv) two-stage multi-plate setup (16-fold compression −40 µJ, 18 fs, 10% throughput)^26^, (v) two stages of HCF (47-fold compression −170 µJ, 6.1 fs, 35% throughput)^13^, respectively. Regardless of the method employed, the common strategy is to make the propagation “as least nonlinear as possible”^27^, in order to avoid that other detrimental nonlinear effects distort the spectral phase and thus achieve a well-defined output.

Here, we report on a straightforward route to comply with this strategy. It has the potential to be scaled to tens of millijoules pulse energies and unprecedented average power levels of hundreds of Watts. Instead of breaking down the whole task into a sequence of small compression stages, we directly employ a single step in which we moderately drive the nonlinear broadening for an extended propagation distance. In this manner, we achieve 33-fold pulse compression in a single stage, by employing a 6-m-long HCF (few-cycle Inc.). We measure 5.1-fs-long pulses, with total transmission efficiency after compression of 70% (670 µJ of energy). Our approach unifies high transmission efficiency and high compression factors, for the generation of ultrashort and energetic few-cycle laser pulses based on Yb amplified systems.

## Experimental Results

We investigated the performance of our single-stage compression scheme for two different requirements. The first aspect considered was maximum compactness. Therefore, we employed a 0.75-m-long HCF with a 400 µm inner diameter. As for the second, complementary aspect - maximum compression performance, we compared 3-m- and 6-m-long HCFs, both with a larger inner diameter of 500 µm. In all cases, the initial input pump pulse condition was the same: 170 fs pulses centered at 1030 nm with an energy of 1 mJ and a repetition rate of 6 kHz (6 W of average power) obtained from a Yb:KGW regenerative amplifier. The pump beam was coupled into the fiber through an AR coated 1-mm-thick FS window, while the output window was uncoated to enable broadband operation. The output beam was collimated by means of an Al-coated concave mirror (f = 1000 mm) and compressed by using custom-made broadband chirped mirror pairs (−50 fs^2^/each bounce)^13^. Different glass windows were used to fine-tune the total dispersion. For all input energies and gas pressures, the total transmission of the 6-m-long system, including chirped mirrors, was 70%. In the case of a 0.75-m-long fiber, the total transmission exceeded 75%. The excellent shot-to–shot RMS energy fluctuations of less than 0.3% at the input remained the same after propagation through the HCF. Finally, the compressed pulses were characterized via a second-harmonic autocorrelator employing a 10-μm-thick BBO crystal (see Section 1 in Supplementary Information).

**Figure 1:**
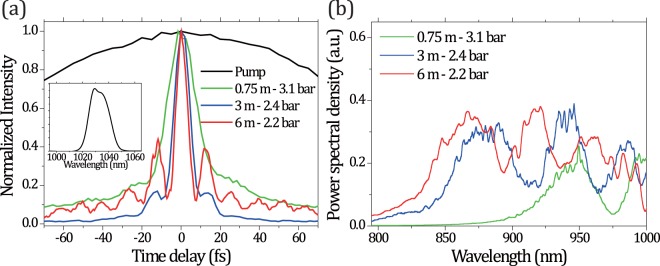
(**a**) Normalized autocorrelation traces of the input pump pulse (170 fs) and the compressed pulses after the 0.75-m-, 3-m-, and 6-m-long Ar-filled HCFs (FWHM: 14, 7, and 5.1 fs, respectively). The total GDDs required in each case are −467.2, −246.9, and −338.6 fs^2^ considering both chirped mirrors and the additional glass windows for fine-tuning. (inset) Pump pulse spectrum. (**b**) NIR-visible spectra for each of the three fiber lengths at optimum pressure conditions.

The experimental comparison of the three fiber lengths confirms that lower instantaneous nonlinearities over an extended propagation distance yield a higher compressibility (Fig. 1a). Nonetheless, even the short 0.75 m fiber enabled a significant compression down to 14 fs FWHM at a static Ar pressure of 3.1 bar. The autocorrelation trace is shown as the green curve in Fig. 1a, together with the one for the 170 fs input pulse (black curve). This is the maximum pressure level for which we can assume the spectral broadening being dominated by SPM (i.e., ionization does not play a significant role yet), since the critical power for self-focusing is 5.1 GW while the input one is 4.7 GW. To move on from here, the only way to further push the compression without compromising stability, transmission or spatial beam quality is to decrease the gas pressure while compensating the lower nonlinearity by a longer propagation distance (Fig. 1b).

In the following, we summarize the physical dependencies behind optimal HCF operation and demonstrate that our route for pushing nonlinear pulse compression is governed by “linear” scaling arguments. For the sake of simplicity, we assume that the pulse compression factor is directly connected to the spectral broadening and thus equal to *Δω*_*in*_*/Δω*_*out*_. This condition is fulfilled as long as the input pulse duration is close to be transform limited. In the case of pure SPM, it can be shown that the spectral broadening is proportional to the accumulated B-integral^10,28^:

1 $$B={k}_{0}{\int }_{0}^{L}{n}_{2}I(z)dz$$

where *k*_0_ is the wave number (*k*_0_ = *2π/λ*_0_), *n*_2_ the nonlinear index of the gas related with the third-order Kerr term, *L* the medium length and *I* the intensity. Treating the noble gas as an ideal gas, where *n*_2_ is directly proportional to the gas pressure *p*, changes Eq. 1 to $B \sim L{n}_{2}pI$, where *n*_2_ is, in this case, the nonlinear index at atmospheric pressure (1 bar). This relation is strictly valid only in the absence of losses. Starting from Eq. 1, we can estimate the ratio of the pulse compression factors (*F*_*comp*_) for two different HCF scenarios:

2 $${F}_{comp}=\,\frac{{L}_{b}{n}_{{2}_{b}}{p}_{b}{I}_{b}}{{L}_{a}{n}_{{2}_{a}}{p}_{a}{I}_{a}}=\,\frac{{L}_{b}\,{p}_{b}{(I{D}_{a})}^{2}}{{L}_{a}\,{p}_{a}\,{(I{D}_{b})}^{2}}\,\,=\frac{3\times 2.4\times {400}^{2}}{0.75\times 3.1\times {500}^{2}}=1.98$$

The right hand side of Eq. 2 is a simplification and valid if the laser input parameters and gas type remain the same, where the intensity can be related to the square of the HCF inner diameter (ID). Inserting the experimental values for the 0.75-m- and 3-m-long HCFs yields an expected compression improvement of 1.98. The shortest FWHM duration obtained for the 3-m-long fiber was 7 fs, displayed as the blue curve in Fig. 1a. The experimentally achieved value for *F*_*comp*_ = 14/7 = 2 shows good agreement with the calculated one based on Eq. 2. Since Eq. 2 is true for pure SPM only, this agreement evidences that the spectral broadening is indeed dominated by SPM. Again, we note that a further increase of pressure in the case of the 3-m-long fiber did not yield shorter pulses but resulted in lower stability or transmission, indicating that other nonlinearities such as self-focusing or ionization become significant under such conditions^29^.

The final step to further increase the compression factor was to extend the fiber length to the limit of the available optical table space. Even though the 6-m-long fiber together with input/output coupling optics requires about 7.5 m of length, the width of the HCF setup can be kept below 10 cm. In this manner, a very short pulse duration of 5.1 fs (for 2.2 bar Ar; Fig. 1a, red curve) was obtained in a single compression step, starting from a 170 fs input. This duration corresponds to 1.5 optical cycles at a center wavelength of 1030 nm. Evaluating the scaling according to Eq. 2, one would expect a compression ratio of 3.63 compared to the case of the 0.75-m-long fiber. The experimental factor of 14/5.1 = 2.75 is about 24.4% less than expected. However, for the 6-m case, linear loss starts to play a role in the broadening effect. More precisely, the numerator of Eq. 2 should be multiplied by a factor $(T-1)/\mathrm{ln}\,T$, where *T* is the power transmission of the fiber. Assuming *T* = *0.75* in the 6-m case, the expected broadening results to be 3.63 × 0.87 ~ 3.1, closer to the experimental value. We underline that linear loss represents the main limiting factor to the effectively usable fiber length and, therefore, the achievable spectral broadening. On one side, the product $I\cdot {n}_{2}\cdot p$ should be kept as low as possible to avoid higher order nonlinear effects and ionization but, on the other side, the length of the fiber cannot be increased indefinitely due to linear loss. Therefore, a trade-off between core diameter and fiber length should be found according to the input pulse conditions, in order to ensure optimal compression.

Since the extreme case of 33-fold pulse compression down to the single-cycle regime with 70% overall transmission is the most striking result of our investigation, we characterized more thoroughly the 6-m HCF operation conditions. First, we investigated the spatial beam quality, which turned out to be very good despite the high broadening factor. The excellent mode quality and the absence of spatial chirp after HCF broadening are displayed in Section 2 of Supplementary Information.

**Figure 2:**
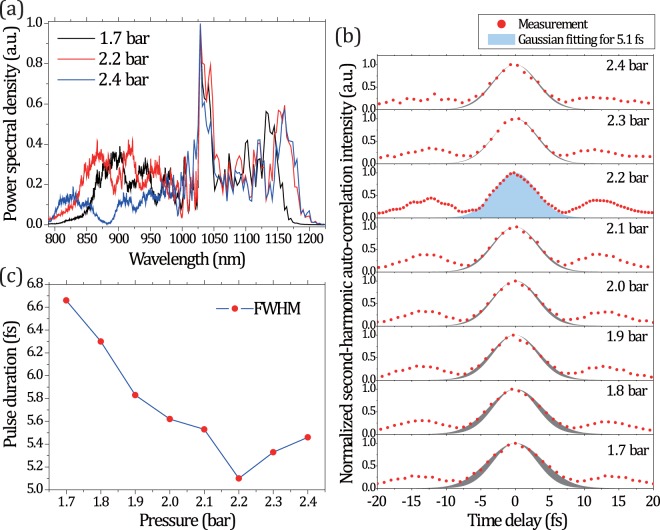
(**a**) Pressure dependent output pulse spectra for the 6-m-long HCF. (**b**) Second harmonic autocorrelation traces corresponding to the minimum pulse durations achieved from 1.7 to 2.4 bar. Each Gaussian fitting curve is compared with the minimum pulse duration fitting (5.1 fs at 2.2 bar) by area subtraction (gray area). (**c**) Compressed pulse durations as a function of the Ar pressure, in terms of FWHM derived from the Gaussian fittings.

Next, we studied the role of gas pressure on the achievable minimum pulse duration. Figure 2a displays the spectral evolution as a function of pressure. At the optimum operation point of 2.2 bar, the spectrum spans over 400 nm, from about 800 nm to 1200 nm. The overall symmetry of the output spectrum and the “well-behaved” spectral modulation support the assumption that SPM plays a major role in the nonlinear propagation even for such an extraordinary broadening.

We also characterized the output pulses both temporally and spectrally, for different pressure values. As expected, incremented pressure enhances the Kerr nonlinearity of the gas, thus initially promoting spectral broadening. However, a further pressure increase beyond 2.3 bar triggers additional nonlinear effects, which in turn impose a complex spectral phase over the pulse envelope that cannot be simply compensated with standard chirped mirrors. Higher pressures also lead to gas ionization and the generation of strongly modulated and asymmetric spectra (the onset of such an effect can be seen in the blue curve of Fig. 2a). The temporal shape of the compressed pulses and the corresponding Gaussian fittings in the Ar pressure range from 1.7 to 2.4 bar are shown in Fig. 2b. In each case, the shortest pulse duration was obtained by fine-tuning dispersion with glass windows. When the pressure exceeds 2.2 bar, the GDD cannot be compensated effectively and the temporal shape results to be somehow distorted with longer pulse tails. A clear optimum is visible at 2.2 bar, where the compressed pulse duration decreases down to 5.1 fs, starting from 6.7 fs at 1.7 bar. The duration increases again for higher pressures (Fig. 2c). We can notice that the pulse shoulders increase while reaching the minimum pulse duration. This might be an effect of the remaining phase oscillations in our compression setup that relies on standard chirped mirrors. To realize an ideal compression scenario in the future, we plan to first characterize the uncompressed phase out of the HCF and use this as an input for the design of the multilayer coating.

## Numerical Simulations

**Figure 3:**
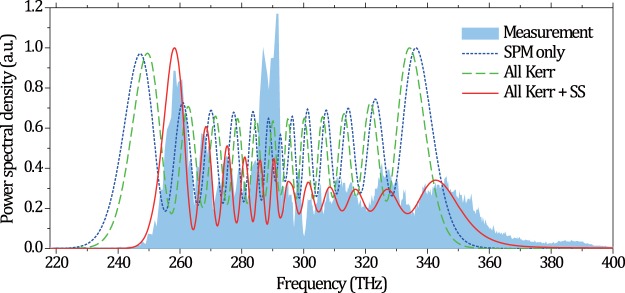
Comparison of the experimentally recorded spectrum (light blue shadow) and the simulated ones for a 6-m HCF at 2.2 bar. The simulations consider SPM only (dotted blue curve), all the Kerr terms only (dashed green curve), and all the Kerr terms including self-steepening (SS) (solid red curve).

Even though the experimental power spectrum for the 5.1 fs pulses (red curve in Fig. 2a) appears rather symmetric when represented as a function of lambda, it is in fact asymmetric when plotted versus frequency due to the Jacobian correction (light blue shadow in Fig. 3). Furthermore, a clear blue shift becomes visible. To gain insight into the origin of this deviation from a pure SPM picture as well as to verify the role of ionization, we compare the experimental output with the results of a 1D + 1 numerical model based on the generalized nonlinear Schrödinger equation (see Section 3 in Supplementary Information). It includes dispersion, all the Kerr terms up to *n*_*10*_, self-steepening, multi-photon absorption and ionization. We note that, since the input peak power (~4.7 GW) is well below the critical value for self-focusing in Ar (≈7 GW at 1030 nm for 2.2 bar)^30^ and because of the excellent spatial homogeneity, the use of a 1D + 1 model can be justified. As can be seen in Fig. 3 (blue dotted curve), pure SPM would lead to a clearly different, totally symmetric output spectrum, with a long wavelength roll off at 220 THz. Including higher order Kerr terms still leads to a symmetric but slightly narrower spectrum. A good agreement is only found when self-steepening is included. Noteworthy, since this temporal re-shaping effect acts on both the trailing and leading edges of the pulses, the spectrum becomes stretched out on the blue side (steepening of the trailing pulse edge) and condensed on the red side (flattening of the leading pulse edge). The center of mass remains about the same, however. Thus, we conclude that SPM plays the dominant role for the vast spectral broadening we observe, while self-steepening, still being a Kerr effect, tends to reshape the power spectrum and the spectral phase. These findings suggest that an asymmetric phase term should be included in the design of chirped mirrors for such extreme pulse compression scenarios^31,32^.

Taking advantage of the good agreement between experiments and theory, we also investigated the energy scalability of our approach, by fixing all experimental HCF parameters and solely changing the type of gas in the simulations (see Section 4 in Supplementary Information). The lower energy limit to reach the same 5.1 fs pulse compression was found to be about 150 µJ, for 2.2 bar of Xe. On the other hand, for 2.7 bar of He, an upper limit of 10 mJ was achieved. Clearly, this two-order-of-magnitude tuning range of input pulse energies can be further extended by using other gas types/pressures or by changing the HCF inner diameter.

## Conclusion

In conclusion, we have demonstrated a straightforward route to direct pulse compression down to the single-cycle regime, starting from 50 cycles. We have shown how a well-structured spectral profile, mainly dominated by SPM, can be achieved by driving the pulse broadening under minimal nonlinearity for an extended propagation distance. This approach allows to channel the pump energy mostly into SPM broadening during propagation, without triggering other additional nonlinear effects that can degrade the spectral phase and prevent a simple post-compression down to the single-cycle. Hence, by means of a 6-m-long HCF, we achieved 33-fold pulse compression of 170 fs pulses, emitted by a commonly available Yb:KGW laser, down to about 5 fs with 70% of overall transmission. This strategy is scalable to tens of millijoules of pulse energies and average powers of hundreds of Watts. Our findings open the path for the direct use of cost-effective, compact and efficient Yb laser technologies in ultrafast science and strong-field laser-driven experiments.

## Electronic supplementary material

Supplementary Information
