# Supplementary material for: Direct compression of 170-fs 50-cycle pulses down to 1.5 cycles with 70% transmission
Source: Sci Rep. 2018 Aug 7;8:11794. doi: 10.1038/s41598-018-30198-y (PMC6081375; doi:10.1038/s41598-018-30198-y)
Supplement: Supplementary file 1 — Supplementary Information [file 41598_2018_30198_MOESM1_ESM.docx]

**Supplementary Information for**

**Direct compression of 170-fs 50-cycle pulses down to 1.5 cycles with 70% transmission**

# Young-Gyun Jeong^1,+^, Riccardo Piccoli^1,+^, Denis Ferachou^1,2^, Vincent Cardin^1,2^, Michael Chini^3^, Steffen Hädrich^4^, Jens Limpert^5,6^, Roberto Morandotti^1,7,8^, François Légaré^1^, Bruno E. Schmidt^2,*^, and Luca Razzari^1,*^

^1^Centre Énergie Matériaux Télécommunications, Institut National de la Recherche Scientifique (INRS-EMT), 1650 Boulevard Lionel-Boulet, Varennes, Québec J3X 1S2, Canada

^2^few-cycle Inc., 2890 Rue de Beaurivage, Montréal, Québec H1L 5W5, Canada

^3^Department of Physics and CREOL, University of Central Florida, Orlando, Florida 32816, USA

^4^Active Fiber Systems GmbH, Wildenbruchstraße 15, 07745 Jena, Germany

^5^Institute of Applied Physics, Abbe Center of Photonics, Friedrich-Schiller-University Jena, Albert-Einstein-Str. 15, 07745 Jena, Germany

^6^Fraunhofer Institute for Applied Optics and Precision Engineering, Albert-Einstein-Str. 7, 07745 Jena, Germany

^7^National Research University of Information Technologies, Mechanics and Optics, 199034 St. Petersburg, Russia

^8^Institute of Fundamental and Frontier Sciences, University of Electronic Science and Technology of China, Chengdu 610054, Sichuan, China

*schmidt@few-cycle.com, razzari@emt.inrs.ca

+these authors contributed equally to this work

# CONTENTS

This document provides supplementary information to “Direct compression of 170-fs 50-cycle pulses down to 1.5 cycles with 70% transmission”. First, further details on the experimental setup are reported in Section 1. In Section 2, the compressed output pulses obtained from a 0.75-m-long HCF are characterized. Section 3 reports the spatial properties of the compressed pulses obtained using a 6 m HCF filled with 2.2 bar of Ar. Section 4 provides the description of the numerical model used to simulate pulse propagation in the gas-filled HCF, as well as a discussion regarding the numerical results and their comparison to the experimental data. Finally, Section 5 presents a numerical investigation on the performance that can be achieved with the proposed compression scheme using other noble gases to handle different input pulse energies.

# Experimental setup

The input pulses were focused down to about 2/3 of the fiber diameter by means of a f = 750 mm convex lens for the 0.75-m-long fiber, and a f = 1000 mm lens for the 3-m- and 6-m-long fibers. After the transmission of the empty fiber was optimized to a value ~ 80%, two metal tubes, incorporating the Ar gas line and vacuum windows, were connected on both sides of the HCF (Figure S1). First, the system was vacuum-pumped, and then Ar gas was slowly injected to achieve a controlled static pressure. The spectrum of the output pulses were measured combining two different spectrometers (Ocean optics covering the region λ < 1100 nm, and Avantes for λ > 945 nm). The recorded spectra were then corrected taking into account the corresponding spectrometer sensitivities, obtained by measuring the spectral intensity of a black body source at a given temperature. Before characterizing the pulse duration, we properly optimized the amount of dispersion added to the HCF output pulses. In case of 6-m-long fiber, after seven bounces on the chirped mirrors, we inserted a 1-mm-thick MgF_2_ window and several 0.15-mm-thick micro-cover glasses to finely adjust the total GDD as well as the third-/second-order dispersion ratio. For instance, at 1.7 bar, we had to add four micro-cover glasses, corresponding to a total thickness of 0.6 mm, to minimize the pulse duration.

# Spatial properties of the output beam

We have investigated the spatial homogeneity of the output beam emerging from the 6-m-long HCF filled with 2.2 bar of Ar. First, we have taken an image of the spatial distribution of the collimated beam profile with a vidicon camera (HAMAMATSU). As it is possible to see in Figure S2a, the output beam profile features a very good spatial quality. Furthermore, we have measured the output spectrum at different positions in the horizontal and vertical directions by scanning the beam across a multimode fiber tip. At each fiber position across the beam profile, we have calculated the percentage of the total intensity (*I*) as the integral of the recorded spectra normalized by the value retrieved at the beam center (i.e., *I* = 100% at the beam center). As illustrated in Figure S2b and c, the spectra are solidly decreasing according to the position along the beam profile (i.e., in terms of collected intensity) and do not show appreciable spatial chirp. We can therefore confirm a homogenous distribution of the spectral components across the central part of beam profile, which is a further advantage stemming from the use of a guiding structure - such as the HCF to achieve pulse compression.

We have also characterized the output pulse duration at the beam center and for the full beam. In both cases, we have found the same pulse duration with differences below the experimental error. This result further underlines the spatial homogeneity of the output pulses coming from the Ar filled HCF.

# Pulse propagation in the Ar-filled HCF

**3.1. Numerical model**

In order to shed light on the compression mechanism, we have accurately modeled the nonlinear pulse propagation through the HCF, following a procedure similar to the one described in Ref.[^1^](#_ENREF_1). We consider a linearly polarized incident electric field $E=Re\left\{ A(z,t)exp[i(k_{0,z}z-\omega_{0}t)] \right\}$ with a central wavelength *λ*_0_ = 1030 nm propagating along the z direction inside the HCF filled with Ar, where $k_{0,z}=2\pi n_{0}/\lambda_{0}$ and $\omega_{0}=2\pi c/\lambda_{0}$ are the wave number and the angular frequency of the carrier wave, respectively. The refractive index *n*(*λ*) is calculated according to the Sellmeier equation of Ar at 1 bar[^2^](#_ENREF_2) and its pressure dependence is given by $n\left( p \right)=\sqrt{1+p(n_{p=1}^{2}-1)}$, where *p* accounts for the gas pressure expressed in bar. The scalar envelope $A(t,z)$ (we denote with $\tilde{A}$ the Fourier transform of $A$) is assumed to be slowly varying in time and along z. Therefore, in the frequency domain, the envelope evolves according to the generalized nonlinear Schrödinger equation[^3^](#_ENREF_3):

$\partial_{z}\tilde{A}=i\tilde{D}\tilde{A}+ik_{0}T\Delta\tilde{n}\tilde{A}-\alpha\tilde{A}/2$ (S1)

The terms in the right-hand side of Equation S1 account, respectively, for dispersion $\tilde{D}\left( \omega\right)=k\left( \omega\right)-k_{0}-k_{1}(\omega-\omega_{0})$, self-steepening $T=1+i\tau_{shock}\partial_{t}$ and $\tau_{shock}\cong1/\omega_{0}$, instantaneous Kerr effects $\Delta\tilde{n}=\sum_{j=1}^{5} n_{2j}\left| A \right|^{2j}$ including the contribution of higher terms up to$n_{10}$, and linear loss $\alpha$. Then, we added to Equation S1 the terms taking into account the ionization of the gas (not shown here for simplicity) as described in Ref.[^4^](#_ENREF_4). The $n_{2j}$ coefficients are related to $\chi^{(2j+1)}$susceptibilities and have been recently reported in Ref.[^5^](#_ENREF_5) for 800 nm. Subsequently, we extrapolate the corresponding values around 1030 nm by using the generalized Miller formulas[^6^](#_ENREF_6), which retrieve the frequency dependence of the $n_{2j}$ coefficients starting from the dispersion of the linear refractive index. The extrapolated nonlinear refractive indexes we used in the simulations are reported in Table S1. The linear loss $\alpha$ is estimated from the experimental transmission measurements such as $\alpha=-\ln T/L$= 0.048 m^-1^, where *T* = 75% accounts for the fiber transmission. The input beam diameter is *2w* = 440 µm, (1/e^2^ of intensity) and *L =* 6 m is the fiber length.

We underline that Equation S1 is valid even for sub-cycle pulses, as shown in Ref.[^7^](#_ENREF_7). Finally, we numerically checked that ionization does not play a major role in the process. According to the PPT theory[^8^](#_ENREF_8), the plasma density for the considered intensities is lower than 10^8^ cm^-3^, thus leading to a refractive index change that is orders of magnitude lower than the Kerr-induced one. We consider an input Gaussian pulse for which the electric field envelope is expressed as Ref.[^3^](#_ENREF_3):

$A(t,0)=\sqrt{\frac{2P_{in}}{\pi w^{2}}}\exp\left( -\frac{t^{2}}{T_{0}^{2}} \right)$ (S2)

where $P_{in}$ is the initial peak power, $w$ the beam radius (at 1/e^2^ of the intensity), and $T_{0}=\Delta t_{FWHM}/\sqrt{2\ln2}$ ($\Delta t_{FWHM}$ is the full width at half maximum of the temporal intensity). The input peak intensity is then calculated as $P_{in}=\sqrt{\frac{2}{\pi}}\frac{E_{in}}{T_{0}}$, $E_{in}$ being the pulse energy. The initial conditions are then chosen to closely match the experimental conditions as reported in Table S2.

Equation S1 is solved with the split-step Fourier method for the 6-m-long fiber. Hence, the dispersion terms are computed in the frequency domain, while both the nonlinear contributions and the self-steepening are treated in the time domain (the latter is solved by using a second-order Runge-Kutta procedure). We ensured that increasing the temporal resolution did not change the numerical results. Note that the input peak power of 4.7 GW is well below the critical power for self-focusing in Ar (≈ 7 GW calculated at 1030 nm and 2.2 bar pressure)[^1^](#_ENREF_1), thus inhibiting a spatio-temporal pulse collapse and fully justifying the use of 1D + 1 model.

**3.2. Results and discussion**

At first, considering the initial conditions reported in Table S2, we simulated the nonlinear pulse propagation through the HCF considering all the Kerr terms and self-steepening. As it is possible to observe in Figure S3, the simulated output spectrum (light blue shadow) and the experimental one (red solid line) are in very good agreement. The third-order Kerr term (*n_2_* in Table S1), is found to be the driving force for spectral broadening[^3^](#_ENREF_3). After 6 m of propagation, the broadened spectrum spans over about 400 nm (from 800 nm to 1200 nm). The maximum phase shift induced by the third order Kerr term can be simply estimated as $\phi_{max}=k_{0,z}n_{2}I_{pk,in}L_{eff}\cong20$ rad[^3^](#_ENREF_3), in very good agreement with the simulated spectral phase (blue dash-dot curve in Figure S3), which shows a well-behaved parabolic trend typical of SPM.

Although SPM plays the major role in the spectral broadening, self-steepening cannot be ignored since it leads to a shift of the spectrum towards shorter wavelengths. In order to track the contribution of the different nonlinear effects, we have successively introduced them in the model and compared the corresponding outcomes. Table S3 summarizes the characteristic lengths *L_x_* of all the considered processes.

As expected, the main nonlinearity driving the pulse propagation (i.e., the process with the shortest characteristic length) is the third order Kerr term. Instead, higher order Kerr terms present a marginal contribution, while gas dispersion remains negligible. Interestingly, a relevant role is played by self-steepening, which physically corresponds to an intensity-dependent group velocity of the pulse[^3^](#_ENREF_3). While the trailing edge becomes steeper and steeper during propagation (corresponding to the generation of blue components near the trailing edge), the leading edge becomes smoother. Therefore, the output spectrum including self-steepening shifts towards the blue when compared to the one obtained under SPM only. Figure S4 illustrates this mechanism showing the power spectral density (dB scale) evolution as a function of the propagation distance, considering only all Kerr terms in a and with self-steepening in b.

This is even more evident considering the output spectra in three cases: model including only SPM, all Kerr terms, and all Kerr terms plus self-steepening; as shown in Figure S5. The difference between only SPM and all Kerr terms is relatively small. Conversely, accounting for self-steepening is the key advance to properly model the experimentally-observed asymmetric spectral shape and its bandwidth extension (also see Figure S3).

Residual discrepancies could originate from the approximation in the steepening term $\tau_{shock}\simeq1/\omega_{0}$. In particular, it has been demonstrated that for quite a broad spectrum, $\tau_{shock}$ has to be corrected as $\tau_{shock}\simeq\frac{1}{\omega_{0}}-\left. \frac{\partial ln(A_{eff}(\omega))}{\partial\omega} \right|_{\omega0}$[^9^](#_ENREF_9), where $A_{eff}(\omega)$ is the effective area of the mode. This correction induces a change in the spectrum asymmetry and could indeed explain the remaining differences between experiments and numerical results in Figure S3. Moreover, even if self-focusing can be neglected, spatio-temporal couplings, which intrinsically cannot be taken into account in 1D simulations, or the excitation of higher-order transverse modes, cannot be totally ruled out for explaining these discrepancies.

# Spectral broadening in Xenon and Helium

In order to demonstrate the scaling potential of our approach, we have performed further numerical simulations using two different noble gases, Xenon and Helium, since they feature approximatively one order of magnitude higher ($n_{2, Xe}=9.16\cdot{10}^{-23}$ m^2^/W at 800 nm) and lower ($n_{2, He}=5.21\cdot{10}^{-25}$ m^2^/W at 800 nm) Kerr nonlinearities in comparison with Ar, respectively. The higher order Kerr terms are obtained from Ref.[^4^](#_ENREF_4). The (linear) refractive index $n(\lambda)$ is evaluated according to the Sellmeier equation of Xenon[^10^](#_ENREF_10)^,^[^11^](#_ENREF_11) and Helium[^12^](#_ENREF_12) at 1 bar, while their pressure dependence was retrieved with the same relation shown before for Ar. For this investigation, we varied the input pulse energy or pressure in order to achieve the same amount of spectral broadening (i.e., the same transform-limited pulse duration) obtained with Ar. All the other parameters (i.e., fiber length, input pulse duration, mode-field diameter) remained the same as for the 6-m-long HCF experiment. Figure S6 shows the simulation results, retrieved considering the full model (all the Kerr terms, as well as the self-steepening contribution). As it is shown, our scheme remarkably allows to achieve the same amount of spectral broadening (i.e., the same potential pulse compression) for input pulse energies ranging within two orders of magnitude (~100 μJ – 10 mJ), by simply changing the gas and finely tuning the pressure within 2-3 bars. More in detail, the simulations show that 0.15 mJ pulses can be broadened by employing Xenon at a pressure of 2.2 bar, see Figure S6a. On the other hand, 10 mJ pulses can be broadened by employing 2.7 bar pressure of Helium (Figure S6b).

As it is possible to observe in Figure S6a, in order to achieve the same spectral broadening starting with only 0.15 mJ of pulse energy, we need to strongly increase the nonlinearity of the system by using Xenon, thus leading to a more irregular spectrum. On the contrary, in the case of high-energy pulses (e.g., 10 mJ) the nonlinearity of the system has to be reduced (i.e., using Helium instead of Ar) (Figure S6b). This behavior further demonstrates the key idea which underlines single-pass compression: moderately driving the nonlinearity, in order not to give rise to other detrimental nonlinear effects which degrade both the spectral amplitude and phase. These results well-demonstrate how almost two orders of magnitude of pulse energies can be simply handled in this scheme, just by changing the gas type and tuning the pressure level.

# References

1. Béjot, P., Schmidt, B.E., Kasparian, J., Wolf, J.P. & Legaré, F. Mechanism of hollow-core-fiber infrared-supercontinuum compression with bulk material. *Phys. Rev. A* **81**, 063828 (2010).

2. Weber, M.J. Handbook of Optical Materials (CRC Press, Boca Raton, FL, 2003).

3. Agrawal, G.P. Nonlinear Fiber Optics (Academic Press, Waltham, MA, 2013).

4. Brée, C. Nonlinear Optics in the Filamentation Regime (Springer, Berlin, 2012).

5. Hauri, C.P. *et al.* Generation of intense, carrier-envelope phase-locked few-cycle laser pulses through filamentation. *Appl. Phys. B* **79**, 673-677 (2004).

6. Ettoumi, W., Petit, Y., Kasparian, J. & Wolf, J.P. Generalized Miller Formulæ. *Opt. Express* **18**, 6613-6620 (2010).

7. Genty, G., Kinsler, P., Kibler, B. & Dudley, J.M. Nonlinear envelope equation modeling of sub-cycle dynamics and harmonic generation in nonlinear waveguides. *Opt. Express* **15**, 5382-5387 (2007).

8. Perelomov, A.M., Popov, V.S. & Terent'ev, M.V. Ionization of Atoms in an Alternating Electric Field *J. Exp. Theor. Phys.* **23**, 924-934 (1966).

9. Kibler, B., Dudley, J.M. & Coen, S. Supercontinuum generation and nonlinear pulse propagation in photonic crystal fiber: influence of the frequency-dependent effective mode area. *Appl. Phys. B* **81**, 337-342 (2005).

10. Bideau-Mehu, A., Guern, Y., Abjean, R. & Johannin-Gilles, A. Measurement of refractive indices of neon, argon, krypton and xenon in the 253.7–140.4 nm wavelength range. Dispersion relations and estimated oscillator strengths of the resonance lines. *J. Quant. Spectrosc. Ra.* **25**, 395-402 (1981).

11. Leonard, P.J. Refractive indices, Verdet constants, and Polarizabilities of the inert gases. *Atom. Data Nucl. Data* **14**, 21-37 (1974).

12. Mansfield, C.R. & Peck, E.R. Dispersion of Helium. *J. Opt. Soc. Am.* **59**, 199-204 (1969).


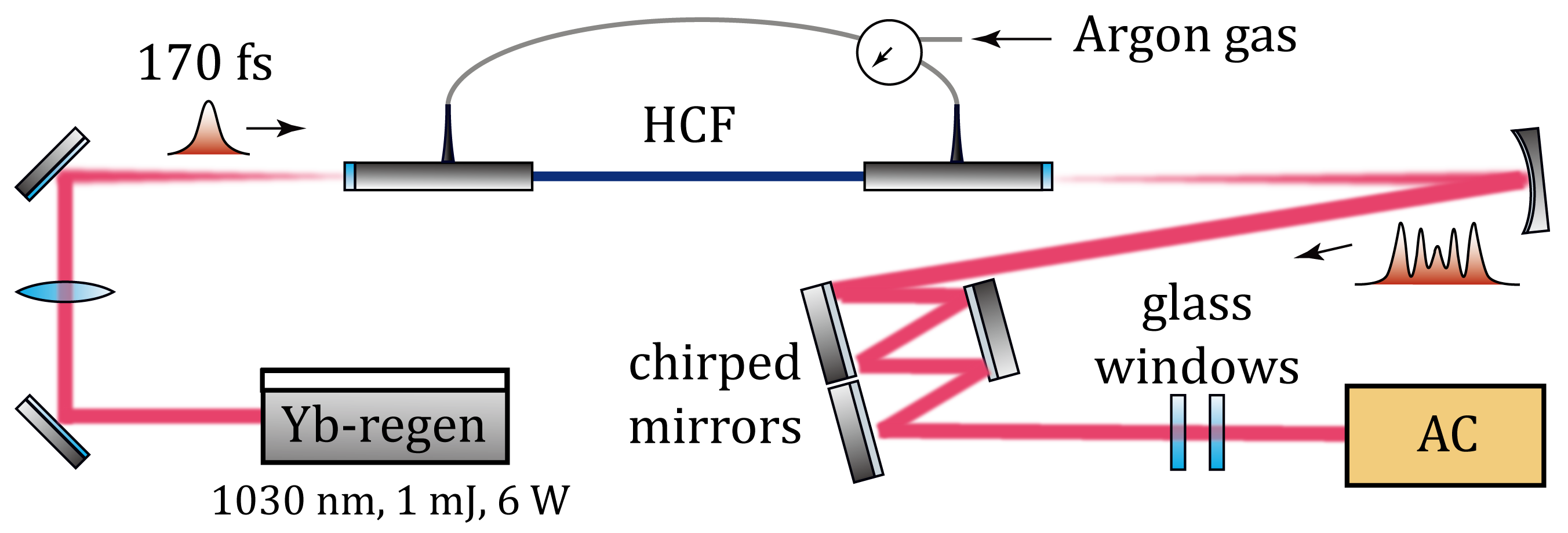


**Figure S1.** Experimental setup.

**Figure S2.** a) Camera image and cross-sectional intensity profiles of the output beam emerging from 6 m HCF filled with 2.2 bar Ar. White lines represent the integrated intensity profile along the horizontal and vertical directions. b) Spectra of the output pulses for different positions along the beam profile in the horizontal, and c) vertical directions starting from the beam center for the NIR-visible region. The corresponding integrated spectral intensity, *I*, is normalized by the value at the beam center.

**Figure S3.** Comparison between the output power spectral density simulated considering all the Kerr terms and self-steepening (light blue shadow) and the spectrum recorded experimentally (red solid line). The dashed blue line corresponds to the simulated spectral phase (right axis).

(b)

(a)

**Figure S4.** Power spectral density (dB scale) evolution as a function of the propagation distance considering a) all Kerr terms, and b) including self-steepening.

**Figure S5.** Power spectral density calculations (solid lines) considering only SPM (orange), all Kerr terms (red), and including self-steepening (SS) (blue). Dash-dot lines represent the simulated spectral phase (right axis) of each spectrum.

(b)

(a)

**Figure S6.** Simulated power spectral densities (blue solid lines) and phases (red dash-dot lines) of the output pulse from a 6-m-long HCF filled with a) 2.2 bar of Xenon, and b) 2.7 bar of Helium. The input pulse energies are 0.15 mJ and 10 mJ, respectively.

| *n_2_* | *n_4_* | *n_6_* | *n_8_* | *n_10_* |
| --- | --- | --- | --- | --- |
| 10^-24^ m^2^ W^-1^ | 10^-42^ m^4^ W^-2^ | 10^-58^ m^6^ W^-3^ | 10^-75^ m^8^ W^-4^ | 10^-94^ m^10^ W^-5^ |
| 9.87 *p* | -3.63 *p* | 3.89 *p* | -1.64 *p* | 8.45 *p* |

**Table S1.** Nonlinear indexes of Ar at 1030 nm.

| *E_p,in_* [mJ] | *∆t_FWHM_* [fs] | *w* [µm] | *P_pk,in_* [GW] |
| --- | --- | --- | --- |
| 0.85 | 170 | 220 | 4.7 |

**Table S2.** Initial conditions used in the model.

| Effect | Characteristic length | Distance [m] |
| --- | --- | --- |
| Kerr 3^rd^ order | $\left\vert c/(\omega_{0}n_{2}\left\vert A_{pk} \right\vert^{2}) \right\vert$ | 0.77 |
| Kerr 5^th^ order | $\left\vert c/(\omega_{0}n_{4}\left\vert A_{pk} \right\vert^{4}) \right\vert$ | 33.83 |
| Kerr 7^th^ order | $\left\vert c/(\omega_{0}n_{6}\left\vert A_{pk} \right\vert^{6}) \right\vert$ | 5.10 |
| Kerr 9^th^ order | $\left\vert c/(\omega_{0}n_{8}\left\vert A_{pk} \right\vert^{8}) \right\vert$ | 19.56 |
| Kerr 11^th^ order | $\left\vert c/(\omega_{0}n_{10}\left\vert A_{pk} \right\vert^{10}) \right\vert$ | 615.66 |
| Kerr (full) | $\left\vert c/(\omega_{0}\sum_{j=1}^{5} n_{2j}\left\vert A_{pk} \right\vert^{2j} \right\vert$ | 0.70 |
| Self-steepening | $\left\vert cT_{0}/(\omega_{0}\sum_{j=1}^{5} n_{2j}\left\vert A_{pk} \right\vert^{2j} \right\vert$ | 29.62 |
| Dispersion | $\left\vert T_{0}^{2}/\beta^{(2)} \right\vert$ | 598.13 |

**Table S3.** Characteristic lengths of the different processes taking place during the propagation.
